# Factors associated with knowledge of diabetic retinopathy among adults with diabetes on follow-up care at public hospitals in Addis Ababa, Ethiopia: an institution-based cross-sectional study

**DOI:** 10.3389/fcdhc.2025.1527143

**Published:** 2025-05-19

**Authors:** Feven Dinsa, Fekadu Aga, Debela Gela

**Affiliations:** ^1^ Department of Nursing, School of Nursing and Midwifery, College of Health Sciences, Wollega University, Nekemte, Ethiopia; ^2^ Department of Nursing, School of Nursing and Midwifery, College of Health Sciences, Addis Ababa University, Addis Ababa, Ethiopia

**Keywords:** knowledge, diabetic retinopathy, diabetes mellitus, adult patient, education

## Abstract

**Background:**

Diabetic retinopathy (DR) is the leading cause of vision loss among adults with diabetes aged between 20 to 70 years. Lack of knowledge about Diabetic retinopathy is one of the reasons for treatment delays, which can lead to the development of sight-threatening DR. The aim of this study is to assess knowledge of diabetic retinopathy and associated factor among adults with diabetes mellitus at public hospitals in Addis Ababa, Ethiopia.

**Methods:**

An institution-based cross-sectional study was conducted at five public hospitals in Addis Ababa from 27 February to 27 March 2023.Systematic random sampling was used to select 421 diabetes patients. Data was collected using a pretested interviewer-administered questionnaire using Kobo Collect version 2022.4.4. The data was then exported to SPSS version 27 for cleaning and analysis. Multiple linear regression analysis with a p-value < 0.05 and corresponding 95% confidence interval (CI) was used to identify factors associated with knowledge of DR.

**Results:**

The respondents’ mean percentage score for DR knowledge was 61.42% ± 28.75%. Longer years lived with diabetes (B = 0.157, p = 0.001) and older age (B = 0.022, p = 0.044) were associated with better DR knowledge score, whereas having no formal education (β = -0.166, p = 0.001) and secondary school education (β = -0.165, p = 0.001) compared to the other groups had lower DR knowledge score.

**Conclusion:**

Longer years lived with diabetes, older age and higher educational level were factors associated with better knowledge of DR. Therefore, health professionals should target newly diagnosed, relatively younger patients and less educated DM patients when providing diabetes self-management education related to DR.

## Introduction

Diabetes mellitus (DM) has emerged as one of the most serious and prevalent chronic diseases of our time, leading to life-threatening, disabling, and costly complications (1). The most common ocular complication of diabetes is diabetic retinopathy (DR) (2, 3). The number of adults with diabetic retinopathy worldwide was estimated to be 103.12 million in 2020, with a projected increase to 160.50 million by 2045 (4). Across Africa, diabetic retinopathy (DR) is a significant health concern, affecting an estimated 35.9% of individuals with diabetes. This prevalence is higher than that observed in North America and the Caribbean, where it is approximately 33.3%.

A systematic review conducted in Ethiopia identifies diabetic retinopathy as a significant microvascular complication, demonstrating the growing impact of diabetes complications in the country (5). The prevalence of diabetic retinopathy in Ethiopia is 19.48% (6). Studies show the incidence of DR is still high after starting treatment among DM patients in Ethiopia. The incidence of DR among treated DM patients from 2020 to 2023 was 25.43% (7). Co-morbid hypertension, poor glycemic control, and prolonged duration of diabetes were all determinants of DR according to the study conducted in Ethiopia (8).

Diabetic retinopathy (DR) is the leading cause of vision loss among adults with diabetes aged between 20 to 70 years (3, 4, 9). In addition to physical disabilities such as blindness, DR can lead to psychological distress, financial challenges, and social isolation (10, 11). Studies also indicate that patients with severe DR experience lower health-related quality of life (HRQoL) scores (11, 12).

Regular eye check-ups and early treatment can prevent the aforementioned consequences of DR. The most crucial factor in the success of any screening program is community awareness. Patients with diabetes who are knowledgeable about the complications of DM, particularly DR, are more likely to engage in proactive health behaviors (13). A lack of knowledge about DR is one of the reasons for treatment delays, which can lead to the development of sight-threatening DR (14). A study reported that patients with a good knowledge of DR were more likely to engage in regular eye check-up practices (15). Having awareness and knowledge about DR also plays a significant role in its overall management and the prevention of severe visual impairment (16).

Different factors can be associated with the knowledge of DR among patients with diabetes. Studies have indicated that a higher educational status (17, 18), urban residence (19, 20), long duration of DM (21, 22), and previous eye disease (19, 20) were positively associated with knowledge of DR. Understanding the level of knowledge about diabetic retinopathy, as well as it’s associated factors, are crucial for designing and implementing effective preventive and treatment interventions.

A subgroup analysis in a study conducted in Ethiopia found an alarmingly high prevalence of diabetic retinopathy in Addis Ababa, which was 35% (23). This shocking result highlights the importance of addressing any knowledge gaps concerning this vision-threatening condition among diabetes patients in Addis Ababa. To successfully address this enormous burden in Addis Ababa, assessing the level of knowledge about DR is crucial. Therefore, this study aimed to assess the level of knowledge regarding DR and associated factors among DM patients receiving follow-up care at public hospitals in Addis Ababa.

## Material and methods

### Study design and period

An institution-based cross-sectional study was conducted in Addis Ababa, the capital city of Ethiopia, which has approximately 6.6 million inhabitants (24). Five of the 14 public hospitals in Addis Ababa were included in the study based on the presence of specialized diabetes care units and a high volume of patient flow. The study was conducted from February 27, 2023, to March 27, 2023.

### Eligibility criteria

All adult patients (18 years and older) with diabetes mellitus (DM) who were receiving follow-up care and had provided consent to participate were included in the study. Patients with DM who were critically ill or had been diagnosed with a severe mental health problem were excluded.

### Sample size and sampling procedure

The sample size was determined using a single population proportion formula, assuming a standard normal distribution (Z) at a 95% confidence level, a margin of error (d) of 5%, and a 47.4% proportion of good knowledge regarding DR among diabetic patients, as reported in a study conducted at Debark Hospital in northwestern Ethiopia in 2021 (20).


$$n\;=\;\frac{{\left(z\frac{\alpha }{2}\right)}^{2}*p\left(1-p\right)}{d^{2}}$$

$$n\;=\;\frac{{\left(1.96\right)}^{2}*0.47\left(1-0.47\right)}{{\left(0.05\right)}^{2}}$$

n= 383

Considering a 10% non-response rate, the final sample size was 421.

Five public hospitals were selected from a total of 14 hospitals located in Addis Ababa. The sample size was proportionally allocated to each hospital based on client flow, which was determined by the average number of clients who received follow-up care at each facility during the month preceding the data collection period. Systematic random sampling was employed to select study participants from the chosen public hospitals. The interval for participant selection was calculated using the formula Kth value = N/nf, where Kth represents the interval at which respondents were interviewed, N denotes the expected total number of diabetic patients per month for all the selected hospitals, and nf indicates the calculated final sample size.

**Figure 1:**
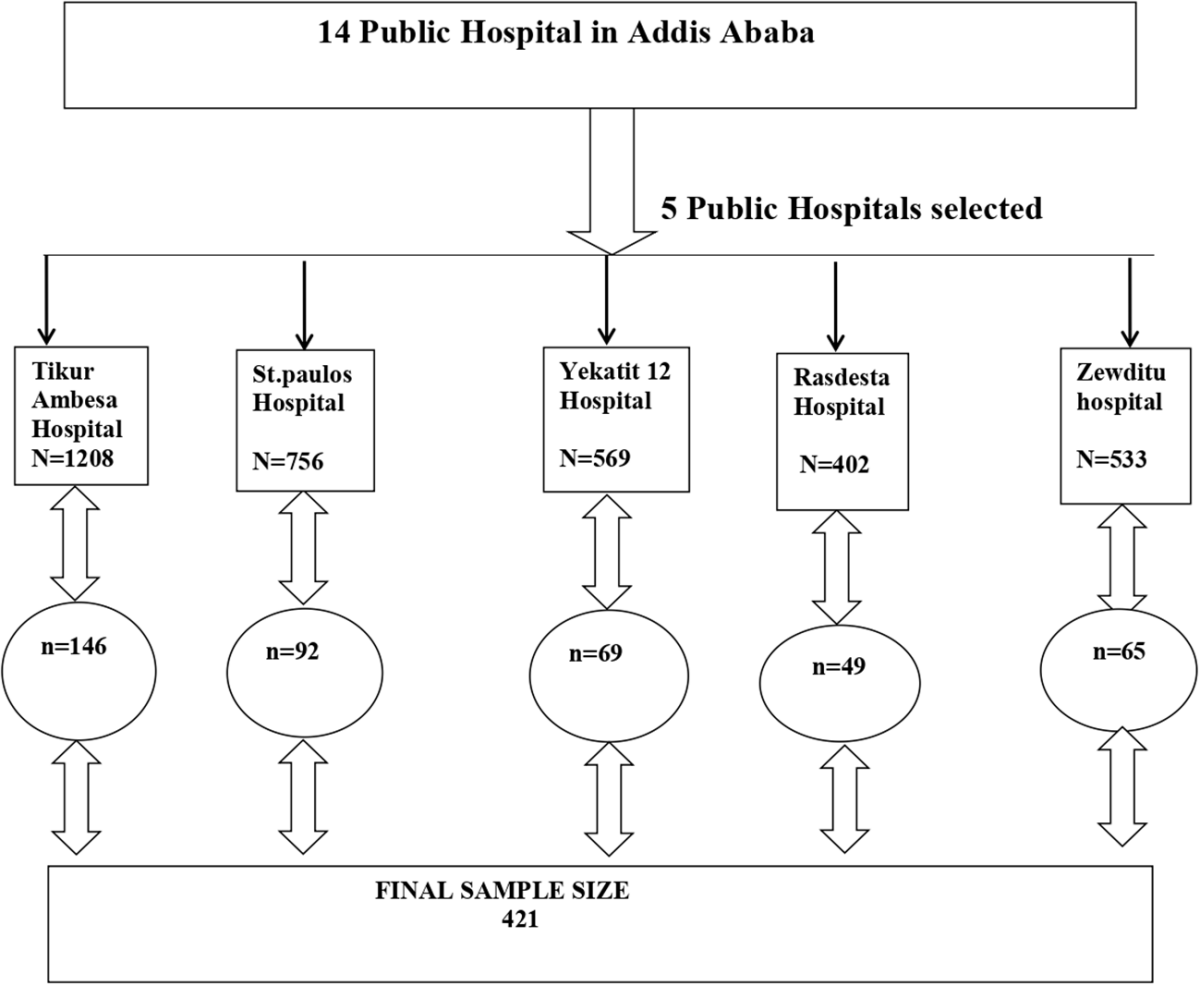
Schematic representation of sampling procedure in selected public hospitals.

The total number of diabetic patients across the five selected hospitals over the past month was 3,468(As indicated in **Figure 1**, Tikur Ambesa Hospital recorded 1,208 patients, St. Paul Hospital had 756, Yekatit 12 Hospital, Ras Desta Hospital, and Zewditu Hospital served 569, 402, and 533 Diabetes patients, respectively, in the month preceding data collection). Therefore, Kth value is calculated as N/nf = 3468/421 = 8. Therefore, using the order of patients’ medical records registered during follow-up appointments as a sampling frame, the first participant was randomly selected from the range of 1 to 8. Subsequent participants were chosen at intervals of 8 until the desired sample size was achieved. The details of the sampling procedure from each hospital was illustrated in **Figure 1**.

### Data collection instrument and procedure

The interviewer-administered questionnaire was adapted from published studies (17, 20, 25). The questionnaire consisted of three parts: a 9-item socio-demographic questionnaire, a 7-item clinical profile, 12 multiple-choice questions to evaluate knowledge of DR, and a single item inquiring about the source of information regarding the impact of DM on the eyes.(online supplemental file 1). The twelve multiple-choice items designed to assess knowledge of DR were categorized into several dimensions, including the definition of DR, risk factors associated with DR, treatment options for DR, and the schedule for eye checkups for individuals with DM. Each correctly answered item was assigned a value of 1, while incorrectly answered items received a value of 0. Knowledge of DR was quantified by calculating the proportion of correctly answered items. The total knowledge score ranged from 0 to 12, with higher scores indicating a better knowledge of DR. The questionnaire was initially translated from English to Amharic and subsequently back-translated to English by various bilingual language experts to ensure the accuracy of the translation. The questionnaire was mounted on Kobo Collect application, version 2022.4.4, to facilitate the digital collection and storage of data on the Kobo Toolbox server. Five nurses, each holding a Bachelor of Science degree, were responsible for data collection. The data collectors received two days of training on basic data collection skills and the use of the Kobo Collect application. The questionnaire was piloted with 5% of the total sample size (21 DM patients) at Alert Hospital(not included in the main study). This was done to ensure the clarity of the items and the consistency in their application two weeks prior to the commencement of data collection for the main study. The internal validity of the instrument was also checked (Cronbach’s alpha = 0.840) which ensures the internal consistency of the instrument.

### Data management and analysis

Data were downloaded from Kobo Collect version 2022.4.4 and exported to SPSS version 27 for analysis. The data were cleaned and checked for completeness. Summary statistics, including the mean with standard deviation (SD), median with interquartile range, and frequency with percentage, were used to summarize the findings. Additionally, text, tables, and graphs were employed to present the data effectively.

Linear regression analysis was employed to examine the association between the dependent and independent variables. Multicategorical independent variables were dummy-coded before running the regression models. The assumptions of linear regression analysis, including normality, linearity, multicollinearity, and homoscedasticity, were thoroughly tested. The normality assumption of multiple linear regressions was checked using a Q-Q plot and histogram of residuals. The histogram of residuals displayed a bell shaped distribution, showing approximate normality. Additionally, in the Q-Q plot the residuals generally showed a close alignment with the normal line, suggesting a fit to normal distribution. To check the assumption of linearity, a scatter plot was used. The plot showed the linear relationship between dependent and independent variable since almost all points of residuals are approximately on the straight line. The assumption of homoscedasticity was evaluated using a plot between standard predictor value and standard residuals. The graphical assessment showed a relatively uniform vertical spreed of the residuals across the range of predicted scores, without systematic widening and funnel shapes and increased/decreased pattern in variance. It indicates that the error term has constant variance. Therefore, it satisfy constant variance assumption approximately. Furthermore, The variance inflation factor (VIF) values were below 10, indicating that there was no multicollinearity.

Variables with a p-value < 0.25 from the initial simple linear regression analysis were included in the multiple linear regression analysis. A p-value < 0.05 and the corresponding 95% confidence interval (CI), was used to determine the statistical significance of the association between the predictor and outcome variables.

## Results

### Sociodemographic characteristics

A total of 421 study participants were included in the study. **Table 1** shows that the majority of the participants were female (53.7%),Orthodox Christians (63.9%), married (78.9%), and urban residents (91%). the mean age of the participants was 53.1 ± 14.92 years.

**Table 1:** Sociodemographic characteristics of the study participants (n = 421).

Variable	Frequency (n)	Percent (%)
Age in years		
18-35	56	13.3
36-50	125	29.7
51-62	110	26.1
≥63	130	30.9
Sex		
Female	226	53.7
Male	195	46.3
Religion		
Orthodox	269	63.9
Muslim	56	13.3
Protestant	88	20.9
Other^a^	8	1.9
Residence		
Urban	383	91.0
Rural	38	9.0
Marital status		
Single	48	11.4
Married	332	78.9
Divorced	15	3.6
Widowed	26	6.2
Educational level		
No formal education	57	13.5
Primary level	124	29.5
Secondary level	114	27.1
Tertiary	126	29.9
Occupation		
Farmer	10	2.4
Daily laborer	5	1.2
Government employed	75	17.8
Housewife	139	33.0
Retired	76	18.1
Merchant	70	16.6
Other^b^	46	10.9
Average monthly income category(ETB)		
≤2000	112	26.6
2001-3577	86	20.4
3578-6500	146	34.7
≥6501	77	18.3

a Includes Catholic and Jehovah's Witness.

b Includes Driver, Students, Non governmental organization employee, Religious worker.

### Clinical profile of the participants

**Table 2** indicates that the majority of participants (74.3%) had type II diabetes. The median duration of diabetes mellitus from the time of diagnosis was 8 years, with an interquartile range (IQR) of 4 to 15 years. Two hundred twenty-nine participants (54.4%) had a history of previous eye disease, and 53.2% reported experiencing visual symptoms.

**Table 2:** Clinical profile of the study participants (n = 421).

Variable	Frequency (n)	Percent (%)
Duration of diabetes		
<10 years	221	52.5
≥10 years	200	47.5
Type of DM		
Type I	108	25.7
Type II	313	74.3
Hypertension		
Yes	227	53.9
No	194	46.1
Family history of DM		
Yes	171	40.6
No	250	59.4
Systemic complications of DM		
Kidney complication	30	7.1
Cardiovascular complication	35	8.3
Other complications of DM^a^	3	0.7
I don’t know	4	1.0
I don’t have any complication	349	82.9
Previous eye disease		
Yes	229	54.4
No	192	45.5
Presence of visual symptoms		
Yes	224	53.2
No	197	46.8

a Includes Diabetic Neuropathy and Foot Ulcer.

### Knowledge of diabetic retinopathy among the study participants

The mean DR knowledge score among the respondents was 7.37 ± 3.45 (out of 12).The percentage standardization of the mean knowledge score was calculated using the following formula:


$$\text{Percentage mean score=}\frac{\text{Actual score}-\text{potential minimum score }}{\text{potential maximum score}-\text{potential minimum score }}*100$$

$$=\frac{7.37-0}{12-0}\;*100$$

= 61.4%

Therefore, The mean percentage score for DR knowledge among the participants was 61.42% of the total expected score, with a margin of error of ± 28.75%.

**Figure 2:**
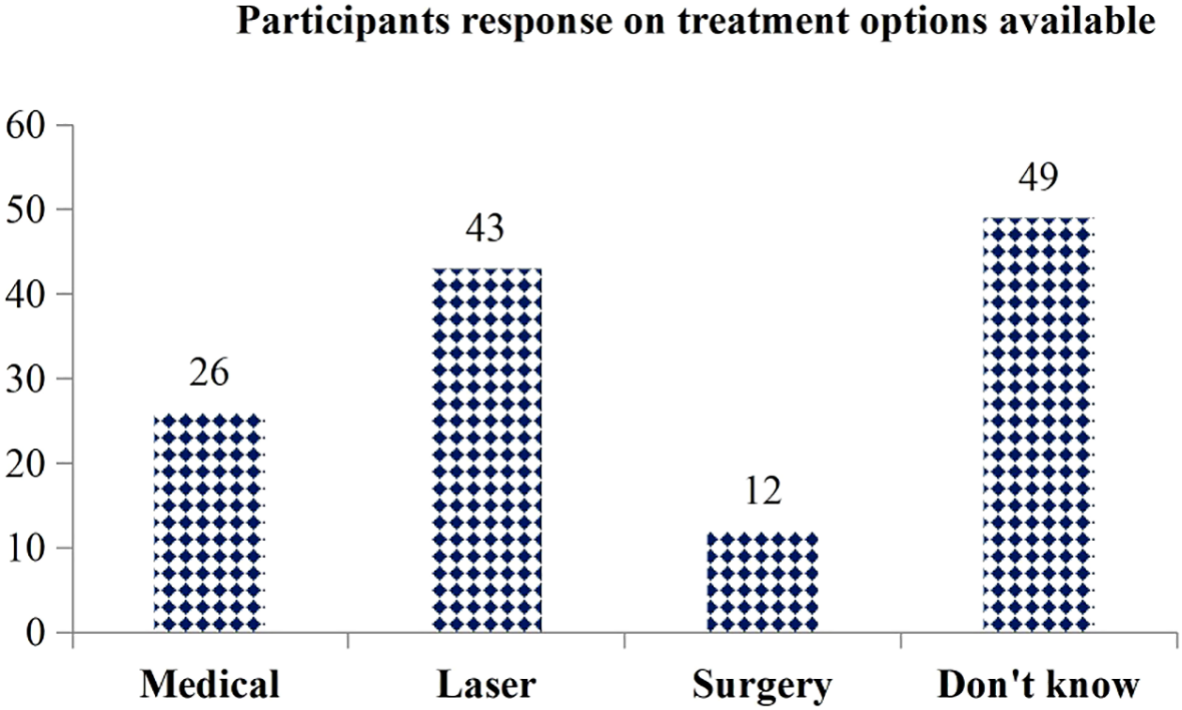
Participant’s response on the treatment options available regarding diabicretinopathy.

The item-by-item analysis presented in **Table 3** below indicates that the majority of study participants (88.8%) are aware that diabetes can affect the eyes and may even lead to blindness (83.1%).Among those who recognized the impact of diabetes on eye health, only 137 (36.63%) were able to correctly name ‘diabetic retinopathy’ as the condition specifically related to diabetes. Furthermore, only 70 participants (18.72%) described the disease process of diabetic retinopathy. Most participants (54%) reported poorly controlled blood sugar as a risk factor for DR. Additionally, 343 participants (81.5%) knew the importance of regular eye checkups (**see** **Table 3**). Of the 124 participants who indicated that DR can be treated, only 43 (34.68%) mentioned laser treatment as a management option for DR (**see** **Figure 2**).

**Table 3:** Knowledge of diabetic retinopathy among the study participants (n = 421).

Items	Frequency (n)	Percent(%)
Does diabetes affect the eye?		
Yes	374	88.8
No	21	5.0
Don’t know	26	6.2
Does it cause blindness?		
Yes	350	83.1
No	11	2.6
Don’t know	13	2.9
What eye condition does diabetes cause?		
Diabetic retinopathy	137	36.6
Cataract	18	4.8
Glaucoma	20	5.3
Don’t know	199	53.2
What is diabetic retinopathy?		
It is the same as a cataract	6	1.6
It is high in sugar in the eye	17	4.5
Change in the blood vessels of the retina	70	18.7
High blood pressure in the eye	15	4.0
Don’t know	266	71.1
What is the risk factor for developing diabetic eye disease?		
Poorly controlled blood sugar	202	54.0
Duration of diabetes	183	48.9
Hypertension	91	24.3
High BMI	24	6.4
Pregnancy	22	5.8
Smoking	20	5.3
Don’t know	55	14.7
Should a person with diabetes check his/her blood pressure?		
Yes	359	95.9
No	2	0.5
Don’t know	13	3.4
Is blood sugar important in preventing blindness from DR?		
Yes	279	74.6
No	6	1.6
Don’t know	89	23.8
Should a person with DM need eye screening?		
Yes	341	91.1
No	8	2.1
Don’t know	25	6.6
How soon after diagnosis need eye screening?		
Immediately	165	48.3
One year after the diagnosis	110	32.2
Five years after diagnosis	10	2.9
Don’t know	56	16.0
Does a patient with DM need a regular eye check-up?		
Yes	343	81.5
No	12	3.2
Don’t know	19	5.0

### Source of information of the participants

**Figure 3:**
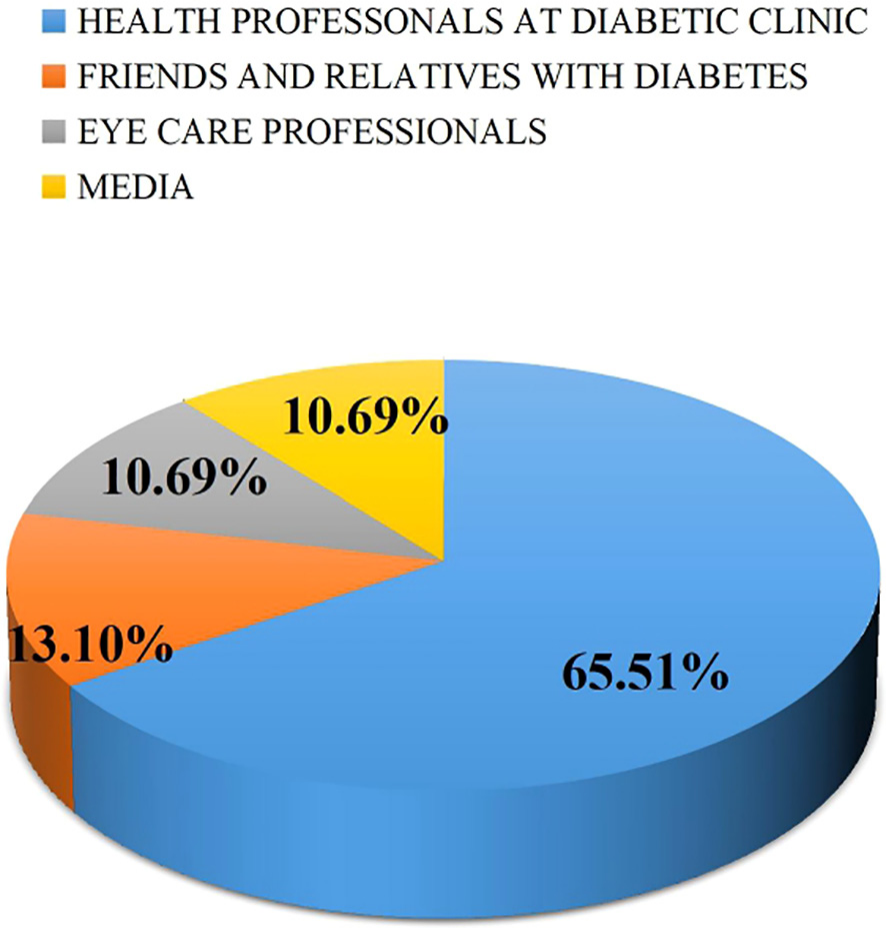
Source of information regarding the effect of diabetes on the eye.

Most of the study participants (65.51%) identified health professionals at the diabetes clinic as their primary source of information regarding the impact of diabetes on the eyes, followed by family members or friends with diabetes. (**Figure 3**).

### Factors associated with knowledge of diabetic retinopathy

To identify the significant candidate variable, a simple linear regression was run for all variables, and then the variables with p-values of 0.25 and below were included in the multiple linear regression analysis. Age, educational status, monthly income, history of eye disease, residence, family history, duration of diabetes, and presence of visual symptoms were candidates for multiple linear regressions.

Multiple linear regression analysis (**Table 4**) identified age, educational status, and duration of diabetes as factors associated with knowledge of diabetic retinopathy. Accordingly, participants who had no formal education were 83.4% less knowledgeable about DR compared to those with other educational categories (β = -0.166, p = 0.001). Similarly, those with a secondary level of education were 83.5% less knowledgeable about DR compared to all the other educational categories (β = -0.165, p = 0.001). For every one-year increase in the duration of diabetes, the knowledge score of DR increased by 0.157 points (B = 0.157; p = 0.001). As age increased by one year, knowledge of DR increased by 0.022 points (B = 0.022; p = 0.044). Over all the variance by 56.7% of knowledge of diabetic retinopathy is due to the effect all predictors as summarized in the final model of the study (R^2^ = 0.567, P<0.000,F=53.59).

**Table 4:** Factors associated with knowledge of diabetic retinopathy among the study participants(n = 421).

Variable	Unstandardized B	Std. Error B	Standardized beta	T	P	95%CI for B	Collinearity	
							Toler	VIF
Age	0.022	0.011	0.094	2.023	0.044	(0.001,0.043)	0.769	1.300
Education								
No formal	-1.668	0.511	-0.166	3.264	0.001	(-2.672,-0.663)	0.641	1.559
Primary	-0.677	0.382	-0.090	1.774	0.077	(-1.427,-0.073)	0.648	1.559
Secondary College	-1.294	0.398	-0.165	3.247	0.001	(-2.077,-0.511) 1	0.625	1.599
Duration of DM	0.157	0.018	0.371	8.525	<0.001	(0.120, 0.193)	0.874	1.144

## Discussion

In this study, 421 participants were included. The primary aim was to assess the level of knowledge regarding diabetic retinopathy and the associated factor among adults with diabetes receiving follow-up care at public hospitals in Addis Ababa. The study revealed a mean knowledge score of 7.37 ± 3.45 regarding diabetic retinopathy. Three hundred seventy-four participants (88.8%) were aware that diabetes can affect the eye. Age, educational status, and duration of diabetes were identified as factors associated with knowledge of diabetic retinopathy.

According to this study, 88.8% of the participants were aware that diabetes can affect the eyes, a figure lower than those reported in studies conducted in Australia (26), Japan (27), and Switzerland (28). This discrepancy may be attributed to the Australian study, which involved members of the Australian Diabetes Association, potentially providing them with access to more current information about diabetes. In contrast, all participants in this study were enrolled regardless of their membership in any diabetes association.

On the other hand, the results of this study are higher than those reported in study conducted in India (29) and northwest Ethiopia (20). This discrepancy may be attributed to variations in the socio-demographic characteristics of the participants. Unlike the current study, the research conducted in India focused on rural areas, where access to information may be limited. Furthermore, a significant portion of the participants in northwest Ethiopia lacks formal education compared to those in the current study.

A systematic review and meta-analysis conducted in Ethiopia reveals a relatively higher prevalence of diabetic retinopathy (19.48%) (6). In comparison, participants in the current study demonstrated slightly above half of the knowledge related questions, achieving an overall score of 61.42%. This might suggest a lack of translation of knowledge into behavior, such as practicing recommended DR screening. Furthermore, indicates that significant efforts are needed to enhance patients’ understanding of diabetes.

Knowledge of risk factors, such as poor control of blood glucose levels, was reported at 54.01%, while awareness of the long duration of DM was 48.93%. These findings are higher compared to studies conducted in India (29) and Ethiopia (20), which reported awareness levels of 42.6% and 36.6%, respectively. This discrepancy may be attributed to variations in the quality and extent of information provided by healthcare professionals regarding risk factors and their consequences. Conversely, the results of this study concerning risk factors, particularly poorly controlled blood glucose, were lower than those reported in a study conducted in Iraq (30).The difference may be due to the fact that the Iraqi study was performed at a teaching eye hospital, where enhanced eye care services and health education are likely to improve knowledge about glucose control and the prevention of diabetic retinopathy.

In this study, 137 participants (36.63%) identified diabetic retinopathy as a complication of DM. This finding is lower than that of a study conducted in Saudi Arabia, which reported a prevalence of 63.5% (31),Additionally, only 70 participants (18.72%) accurately defined diabetic retinopathy in the current study, a figure that is significantly lower than the 55% reported in a study from Bangladesh (17), This discrepancy may be attributed to limited access to information and insufficient media engagement in the present study.

According to this study, 81.5% of the participants recognized the importance of regular eye checkups. This finding is significantly higher than those reported in studies conducted in southern Ethiopia (19), India (29) and Nigeria (32).This discrepancy may be attributed to the fact that the majority of participants in the southern Ethiopia study had no formal education, unlike those in the current study. Many health education programs rely on written materials and presentations, which can pose challenges for individuals with lower literacy levels in effectively engaging with the content. Conversely, the results of the current study are lower than those of a study conducted in Switzerland (28), which reported a rate of 97.5%. This difference may be due to the selection of study participants; the Swiss study included a higher proportion of individuals with pre-existing eye disorders. Those with identified visual impairments are generally more proactive about attending regular examinations, which could explain the significant disparity in awareness levels between the two studies.

This study found that age, educational status, and duration of diabetes were significantly associated with the knowledge score regarding diabetic retinopathy. As participants’ age increased, their knowledge score regarding DR also increased. This finding is consistent with other studies conducted in Saudi Arabia (33) and Iran (34). This association may be attributed to increased healthcare utilization among older adults. As the risk of developing eye diseases escalates with age, older individuals tend to visit healthcare facilities more frequently. These visits provide opportunities for them to acquire knowledge about DR and other eye conditions. However, some previous studies conducted in other regions of Ethiopia and Malaysia (17, 19, 35).

Educational status was another factor significantly associated with the knowledge score of diabetic retinopathy (DR). Individuals with no formal education and those with only a secondary education exhibited lower DR knowledge scores compared to those with tertiary-level education (college level). The findings of previous studies corroborate the results of the current study (17, 36–39). This discrepancy may be attributed to inability of Diabetes patients who are not formally educated or who have only had primary education to adequately obtain and comprehend health information. Lack of education leads to a low literacy level and a reduced ability to conduct independent research on their health concerns. Additionally, the lower DR knowledge scores are more likely due to limited accessibility of health education materials. For those with no formal education, being illiterate prevents them from accessing educational materials (brochures, websites), preventing basic knowledge acquisition about DR. While those with secondary education have some literacy, their makes complex medical information difficult to understand, hindering them to grasp details of DR.

The duration of diabetes is another factor associated with knowledge of DR. As the duration of diabetes increases, so does the knowledge score regarding DR. These findings corroborate those of previous studies conducted in various regions of the world (19, 21, 22, 38). A possible explanation for this trend is that individuals who have lived with diabetes for a longer period may be more motivated and have greater opportunities to receive education about DM and its complications. However, studies conducted in Goa (37), South Indian State (36), and Northwest Ethiopia (17)found no association between the duration of DM and knowledge of DR.

This study suggests that there is still a significant knowledge gap regarding DR among diabetes patients in Addis Ababa. This lack of knowledge contributes to delayed presentation for eye examinations, poor adherence to recommended screening schedules and finally leading to poor self care practice related to eye health.

The clinical relevance of this study is to provide information on the level of knowledge of diabetic retinopathy and associated factors. It enables clinicians (health care professionals) to fill the knowledge gap proactively during diabetes patient consultations and include comprehensive diabetic retinopathy education in to the routine diabetic care. From the perspective of public health, this study helps to reduce diabetic retinopathy treatment cost, ultimately reducing the economic loss due to DR. By filling the knowledge gap, this study can contribute to minimize productivity loss, prevent blindness, and alleviate the burden on the healthcare system.

## Conclusion and recommendation

This study indicates that, on average, participants answered slightly more than half of the knowledge-related questions correctly, achieving a percentage mean score of 61.42%. While this demonstrates some awareness, it also highlights a significant knowledge gap. Therefore, these findings underscore the urgent need for enhanced educational interventions to improve diabetes patients’ awareness and knowledge of diabetic retinopathy. This can be effectively accomplished through targeted, continuous education provided by community health workers, which will not only enhance diabetes patients’ knowledge but also aid in the early detection of diabetic retinopathy. Knowledge of diabetic retinopathy increases with the duration of diabetes and the patient’s age. Conversely, patients with DM who have lower levels of educational attainment tend to have less knowledge about DR.

Healthcare professionals need to conduct thorough assessments of patients’ understanding of DR to enhance their comprehension of treatment and preventive options. Notably, healthcare professionals from diabetes clinics have been identified as the primary source of information for patients regarding the effects of diabetes on the eyes. Therefore, as the main source of information, healthcare professionals should prioritize providing diabetes self-management education related to DR to newly diagnosed patients, younger individuals, and those with lower educational attainment.

### Limitations of this study

The study was limited to public hospitals and did not include diabetes patients receiving follow-up care at private healthcare institutions. Furthermore, it does not determine whether participants’ knowledge of diabetic retinopathy translates into actual health-seeking behavior or the implementation of recommended eye care measures. Another limitation of this study is the questionnaire’s potential for social desirability bias.

## Data Availability

The original contributions presented in the study are included in the article/**Supplementary Material**. Further inquiries can be directed to the corresponding author.
