# Supplementary material for: Factors associated with knowledge of diabetic retinopathy among adults with diabetes on follow-up care at public hospitals in Addis Ababa, Ethiopia: an institution-based cross-sectional study
Source: Front Clin Diabetes Healthc. 2025 May 19;6:1527143. doi: 10.3389/fcdhc.2025.1527143 (PMC12127204; doi:10.3389/fcdhc.2025.1527143)
Supplement: Supplementary file 1 [file DataSheet1.pdf]

Questionnaire (English version)

Part 1: Socio-demographic and socio economic data

S. No	Question	Response
1	Sex	1. Female 2. Male
2	Age in year	-------------
3	Religion	1. Orthodox 2. Muslim<br>3. Protestant 4.Other…………….
4	Residence	1. Urban 2. Rural
5	Marital status	1. Single 2. Married<br>3. Divorced 4. Widowed
6	Educational level	1. Can't read and write 2. Primary (1 -8)<br>3. Secondary 4 .Tertiary (College/University)
7	Occupation	1.Farmer 2.Daily laborer<br>3.Government employed 4.House wife<br>5.Retired 6.Merchant<br>7. Other specify………………………………………………..
8	Monthly income (in Ethiopian birr)	**----------------**

Part 2: clinical profile of the patient

9	How long have you been diabetic?	----------------
10	Type of DM	1.Type-1 2.Type-2<br>3. Don't know
11	Do you have Hypertension?	1.yes 2.No<br>3. Don't know
12	Is there anyone with diabetes in your family?	1. Yes 2. No
13	Which Systemic complications of DM do you have?	1. Kidney complication<br>2. Cardiovascular complication<br>3. Other complication of diabetes<br>4. I don't know<br>5. I don't have any complication of DM
14	Previous history of eye disease in the past	1.yes 2.No<br>3. Don't know
15	Do you have any problem with vision / Do you have any visual symptoms?	1 .yes 2.No

Part 3: Knowledge of Diabetic retinopathy

Serial No,	Question	Response
16	Does diabetes affect the eye?	1.Yes 2.No<br>3.Don't know
17	Can diabetes cause blindness? (If answered "No" to both the above questions #14 and #15, please go to question #27)	1.Yes 2.No<br>3.Don't know

18	What eye condition does diabetes specifically cause in the eyes?	1.Diabetic retinopathy 2.Cataract<br>3. Glaucoma 4. Don't know<br>5. Others…………………
19	What is diabetic retinopathy?	1. It is the same as cataract.<br>2. It is high sugars in the eye.<br>3.It is changes in the blood vessels of the retina due to diabetes<br>4.It is high pressure in the eye<br>5.Don't know
20	What are the risk factors for developing diabetic eye disease? (You can choose more than one answer)	1.Poorly controlled blood sugar 2.Duration of diabetes<br>3.Hypertension 4.High BMI<br>5.Pregnancy 6.Smoking<br>7.I don't know
21	Should a person with diabetes cheek his/her blood pressure?	1.yes 2.No<br>3.don't know
22	Is blood sugar control important in preventing blindness from diabetic Retinopathy?	1.Yes 2.No<br>3.Don't know
23	Is diabetic eye disease treatable? (If answered "No" to the above question, please go to question #23)	1.Yes 2.No<br>3.Don't know
24	What are the treatment options available for diabetic eye disease?(You can choose more than one answer)	1.Medical (Injections inside the eyes) 2.Laser burns inside the eyes<br>3.Surgery 4.don't know
25	Should a person with diabetes mellitus need eye screening?	1.Yes<br>2.No (If answered "No" to this question, please go to question #25)<br>3.Don't know
26	How soon after the diagnosis has been made should that person visit the	1. Immediately after diagnosis of DM<br>2. One year after diagnosis

	specialist eye doctor?	3.Five years after diagnosis<br>4.Other __________________	
27	Does a diabetic patient need a regular eye checkup?	1.Yes 2.No<br>3.I don't know	
28	How did you come to know about DM affecting the eyes?	1.Health professional at the diabetes follow up clinic<br>2.eye care professional's<br>3.Family member/relative/friend with diabetes<br>4. TV, magazines, other media<br>5.Other…………………………..	
